# Endoplasmic Reticulum Stress and Autophagy Are Involved in Hepatotoxicity Induced by Tributyltin

**DOI:** 10.3390/toxics11070607

**Published:** 2023-07-12

**Authors:** Weiqi Liang, Lingling Fu, Mei Feng, Xiaorong Wang, Zhaohui Yun, Jin Xu

**Affiliations:** 1 School of Public Health, Health Science Center, Ningbo University, Ningbo 315211, China; 2 Zhejiang Key Laboratory of Pathophysiology, Health Science Center, Ningbo University, Ningbo 315211, China

**Keywords:** tributyltin, hepatotoxicity, proteomics, endoplasmic reticulum stress, autophagy

## Abstract

Tributyltin (TBT), a common contaminant in aquatic ecosystems, has severe toxic effects on multiple tissues and organs, especially the liver. Previous toxicogenomic analysis has indicated that the main mechanism of TBT-induced hepatotoxicity is related to the activation of the apoptotic pathway. However, the mechanism of action occurring before the activation of apoptosis is still unclear. Herein, we applied proteomic technology to explore the protein expression profile of TBT-treated HL7702 normal human liver cells. The ultrastructural changes in cells were observed by transmission electron microscopy. After low dose (2 μΜ) TBT treatment, activation of the unfolded protein response and endoplasmic reticulum stress were observed; the expression levels of PERK, ATF6, BiP, and CHOP were significantly elevated, and splicing of *XBP1* mRNA was initiated. When the TBT concentration increased to 4 μΜ, the protein levels of Beclin1, Atg3, Atg5, Atg7, and Atg12-Atg5 were significantly elevated, and the protein level of LC3Ⅰ decreased while that of LC3Ⅱ increased, suggesting the activation of autophagy. As the TBT concentration continued to increase, autophagy could not eliminate the damage, and apoptosis eventually occurred. These results indicate novel pathways of hepatotoxicity induced by TBT and provide insights for future studies.

## 1. Introduction

As important raw materials of chemicals, textiles, and pesticides, organotin compounds (OTCs) are extensively applied in industrial and agricultural production [1,2]. Tributyltin (TBT) is a typical type of OTC that was originally used as a decontaminant in hull paints in the marine industry to reduce the buildup of barnacles [3], and later as the main active ingredient in biocides, which are widely used in various industries [3]. However, TBT is regarded as one of the most dangerous anthropogenic contaminants in aquatic ecosystems due to its detrimental effects on both the human health and environment [4,5,6]. TBT is a potent endocrine disruptor and may pose a threat to nontarget marine organisms. As a typical example of such contamination, TBT can bioaccumulate and cause imposex in gastropods, even when the concentration of tin is very low (1–2 ng L^−1^) [7,8]. Consequently, TBT may cause impairment in the growth, development, and even survival of nontarget organisms [9]. After many countries gained an in-depth understanding of the hazards of TBT, various measures were successively taken to restrict or prohibit the application of TBT in paints [3].

Nevertheless, TBT may continue to be applied as a biocide in other industries, for example, as a material or wood preservative [10]. Additionally, due to its strong affinity for sediments and suspended particulates and the relatively high persistence of OTCs in anaerobic environments, sediment is a source through which TBT can be reintroduced into aquatic environments, especially in countries that have not imposed any restrictions [6,11,12]. As a result of their ubiquity in the marine environment, OTCs may accumulate in the food chain and appear in seafood products [13]. The use of fish contaminated with TBT as a food source has been reported to pose a health risk to some extent due to the biomagnification of TBT [14,15]. Furthermore, intensive research has demonstrated that TBT seriously jeopardizes the functions of internal organs and systems in humans and laboratory animals, with effects such as reproductive toxicity, neurotoxicity, immunotoxicity, and hepatotoxicity [2,16,17,18]. As a consequence, the potential impact of TBT on human health is still a concern.

It has been widely reported that TBT can cause cell proliferation inhibition, cell cycle arrest, cytoskeletal disruption, and apoptosis [19,20]. TBT exposure leads to an increased incidence of liver damage and decreases in the metabolic and detoxification abilities of the normal human liver cell line (HL7702 cells) [21]. Existing evidence suggests that the liver is the organ with the highest concentration of TBT in marine mammals [2,15,22,23]. The same trend in TBT accumulation has been shown in human sampling studies [24,25,26]. TBT has also been reported to be hepatotoxic at doses even lower than the proposed human tolerable daily dose [27,28,29]. Considering the accumulation and metabolism of TBT in the liver, it is of great significance to elucidate the mechanism of TBT-induced hepatotoxicity [17].

Although the mechanism of TBT-induced toxicity is complex, it is well-known that apoptosis may play a dominant role [30,31,32,33]. Moreover, our previous toxicogenomic analysis confirmed that activation of the apoptotic pathway might be the major mechanism of TBT-induced hepatotoxicity [20]. However, transcriptomic research alone is insufficient to gain a complete understanding of the complex mechanism, since it only indicates mRNA expression without considering the subsequent translational process. Therefore, in this study, the isobaric tags for relative and absolute quantitation (iTRAQ) technology were applied to explore the effects of TBT on the protein expression profile in HL7702 cells, which may provide new insights into the complex mechanisms of TBT-induced hepatotoxicity.

## 2. Materials and Methods

### 2.1. Chemicals

TBT and dimethyl sulfoxide (DMSO) were obtained from Sigma-Aldrich (St. Louis, MO, USA). Cell lysis buffer, fetal bovine serum (FBS), RPMI 1640 medium, and Western Bright Enhanced Chemiluminescence (ECL) detection reagents were obtained from Thermo Fisher Scientific (Suwanee, GA, USA). An Annexin V/PI apoptosis detection kit was purchased from Multi-Sciences (Hangzhou, China). Anti-Bip (Cat#3177), anti-Calnexin (Cat#2679), anti-Ero1-Lα (Cat#3264), anti-IRE1α (Cat#3294), anti-PDI (Cat#3501), anti-CHOP (Cat#2895), anti-PERK (Cat#5683), anti-eIF2α (Cat#5324), anti-P-eIF2α (Cat#3398), anti-Atg12 (Cat#4180), anti-Beclin-1 (Cat#3495), anti-JNK (Cat#3708), anti-P-JNK (Cat#4668), anti-LC3A/B (Cat#12741), anti-Atg5 (Cat#12994), anti-Atg16L1 (Cat#8089), anti-Atg7 (Cat#8558), anti-Atg3 (Cat#3415), anti-rabbit IgG (Cat#7074), and anti-mouse IgG (Cat#7076) were obtained from Cell Signaling Technology (Beverly, MA, USA). Anti-ATF6 (Cat#ab227830) was purchased from Abcam (Cambridge, UK) and anti-GAPDH was obtained from KangChen Biotech (Shanghai, China).

### 2.2. Cell Culture and TBT Treatment

HL7702 cells were obtained from the Chinese Cell Bank. The cells were cultured in RPMI 1640 medium with 10% FBS at 37 °C in a humidified CO_2_ (5%) incubator. After growth for 48 h, cells were treated with 0, 2, 4, 6, 8, or 10 μΜ TBT for another 2 h.

### 2.3. Cell Proliferation Assay

The methyl thiazolyl tetrazolium (MTT) assay was applied to detect the cell proliferation [34]. Approximately 2 × 10^3^ cells were inoculated into each well of the 96-well cell culture plate and cultured for 48 h. The cells in each group were treated with 0, 2, 4, 6, 8, or 10 μΜ TBT for 2 h, respectively. After removing the TBT-containing medium, 10 μL of 5 mg/mL MTT and 90 μL of fresh medium with 10% FBS were then pipetted into each well and incubated for another 4 h. The MTT-containing medium was subsequently discarded and the purple crystals were completely dissolved in 150 μL of DMSO for 10 min. Finally, the optical density of each well was detected at 490 nm through a microplate reader. The difference in cell proliferation was expressed as the proportion of the proliferation of untreated cells.

### 2.4. Ultrastructure Observation

After treatment with different concentrations of TBT for 2 h, the HL7702 cells were fixed with a 2.5% glutaraldehyde solution for 4 h. Each cell sample was washed with PBS three times, fixed for 1 h with 1% osmium tetroxide, and then postfixed with 2% uranyl acetate for 30 min. Thereafter, the samples were dehydrated with 50%, 70%, 90%, or 100% ethanol and transferred into anhydrous acetone. Each of the above steps took 20 min. Each sample was subsequently treated with an equal ratio mixture of absolute acetone and ethoxyline resin for 2 h, followed by an epoxy resin coating solution for another 2 h. Each sample was cured under a temperature gradient (37 °C to 45 °C) for 24 h, followed by 60 °C for 48 h. Lead citrate and uranyl acetate were used to stain the samples. Then, the samples were examined by a certified histologist using a TECNAI 10 transmission electron microscope (TEM). Three fields were randomly selected from the upper, lower, left, right, and middle sides of the field for observation.

### 2.5. Detection of the Protein Expression Profile Using iTRAQ

The steps of the iTRAQ experiment were as follows. (1) Sample preparation: about 1 × 10^5^ cells were incubated in 25 cm^2^ plates for 24 h and then treated with TBT (0, 2, 6, or 10 μΜ) for an additional 2 h. Then, each sample was completely immersed in 200 μL of protease inhibitor-containing lysis buffer and lysed on ice. The supernatant was collected after ultrasonication for 3 min and centrifuged at 4 °C for 10 min at 12,000× *g*. Cooled acetone was added to each sample and hatched at −20 °C for 2 h. After that, the precipitate was collected and dissolved in cell lysis solution. The BCA method was applied to determine the protein concentration [35]. The proteins in the samples were separated by 12% sodium dodecyl sulfate-polyacrylamide gel electrophoresis (SDS-PAGE) followed by Coomassie brilliant blue staining to determine the quality of the extracted proteins. To reduce the effects of individual differences between the samples, the samples from each group were pooled into two samples. (2) Protein digestion and iTRAQ labeling: the digestion of the protein samples was carried out in accordance with the filter aided sample preparation (FASP) procedure [36]. The experimentally obtained peptide mixture was labeled with a modified eight-plex iTRAQ^®^ reagent (AB Sciex Inc., Framingham, MA, USA). Proteins extracted from HL7702 cells exposed to 0, 2, 6, or 10 μΜ TBT were labeled with eight-plex iTRAQ reagents, respectively. Finally, all samples were mixed before proceeding to subsequent experiments. (3) Peptide fractionation: the AKTA Purifier system fractionated iTRAQ-labeled peptides through strong cation exchange chromatography (GE Healthcare, Little Chalfont, UK) [37] and made preliminary judgments about the peptide samples and separation efficiency based on the chromatogram. (4)LC-MS/MS analysis: experiments were performed on TripleTOF 5600 mass spectrometer (AB Sciex Inc., Framingham, MA, USA). (5) Protein identification and quantification: MS/MS spectra were retrieved with the Protein-Pilot software (version 5.0, AB Sciex, Framingham, MA, USA, 2015) against the *Homo sapiens* database using the Paragon algorithm [20]. Protein identification was performed with the search option: emphasis on biological modifications. The database search parameters were as follows. Cysteine alkylation: iodoacetamide; Digestion: trypsin; Instrument: TripleTOF 5600 (AB Sciex Inc., Framingham, MA, USA); Search effort: thorough; FDR analysis: yes. (6) Bioinformatics analysis: the UniProtKB protein database was used to retrieve the sequences of all differentially expressed proteins (DEPs). The Kyoto Encyclopedia of Genes and Genomes (KEGG) pathway database was then used to further analyze the relevant pathways of the DEPs.

### 2.6. Detection of Endoplasmic Reticulum (ER) Stress

ER stress was detected by analyzing the splicing of x-box binding protein 1 (*XBP1*) mRNA [38]. After treatment with different concentrations of TBT for 2 h, a HP Total RNA Kit (Omega Biotek, Norcross, GA, USA) was used to isolate the total RNA from the HL7702 cells. The HiFiScript cDNA Synthesis Kit (CWbio, Beijing, China) was then used for cDNA synthesis. The PCR amplification was performed using PCR Master Mix (Cwbio, Beijing, China). The primer sequences used for *XBP1* splicing analysis were GAGTTAAGACAGCGCTTGGG and ACTGGGTCCAAGTTGTCCAG [38]. The spliced and unspliced *XBP1* mRNAs were electrophoresed on a 2.5% agarose gel stained with Gel Red.

### 2.7. Detection of Autophagy

The presence of autophagic vacuoles was detected with a CYTO-ID Autophagy Detection Kit (Enzo Life Sciences, Farmingdale, NY, USA) in accordance with the manufacturer’s instructions. HL7702 cells were grown on collagen-coated cover glasses and treated with 0, 2, 4, 6, 8, or 10 μΜ TBT or with chloroquine as the positive control for 2 h. Then, the cells were incubated with Hoechst 33342 Nuclear Stain Reagent and CYTO-ID Green Detection Reagent at 37 °C for 30 min in the dark. All cells were analyzed under a LEICA TCS SP8 confocal microscope. The autophagic rate was estimated by counting cells in five randomly selected areas in duplicate.

### 2.8. Detection of Apoptosis

The Annexin V-FITC Apoptosis Detection Kit (Enzo, New York, NY, USA) was used to detect apoptosis. After treatment with different concentrations of TBT for 2 h, the HL7702 cells were transferred into a microcentrifuge tube. Next, 200 μL of annexin V-FITC mixture with 10 μL of PI and 5 μL of annexin V-FITC were added to each tube. The apoptosis rate was determined with a Beckman Coulter flow cytometer (Beckman Coulter Company, Brea, CA, USA).

### 2.9. Western Blotting

Aliquots of 50 μg protein were isolated by SDS-polyacrylamide gels and transferred to polyvinylidene difluoride (PVDF) membranes. The transferred membranes were blocked at room temperature for 2 h with 5% bovine serum albumin (BSA), and then incubated by incubation medium containing primary antibodies overnight at 4 °C [specific for BiP, Ero1-Lα, Calnexin, PERK, CHOP, IRE1α, P-IRE1α, PDI, Beclin-1, elF2α, P-elF2α (Ser51), JNK1, P-SAPK/JNK (Thr183/Tyr185), LC3I/II, Atg3, Atg5, Atg7, Atg12 (16/55 kDa), or Atg16L1 1:1000]. Subsequently, the membranes were immunoblotted with the corresponding secondary antibodies (1:2000) at room temperature for 2 h and then incubated with ECL reagents. To verify equal amounts of protein, an anti-GAPDH antibody (1:10,000) was used to probe the membranes.

### 2.10. Statistical Analysis

All experiments were repeated at least three times, and the data were presented as the mean ± standard deviation ($\bar{X}\pm S$). Statistical analysis of the relevant data was performed using SPSS 21.0 software, and the differences among groups were analyzed by ANOVA. Statistical significance was determined by *p* values of <0.05.

## 3. Results

### 3.1. TBT Induced Alteration of Cell Proliferation

**Figure 1:**
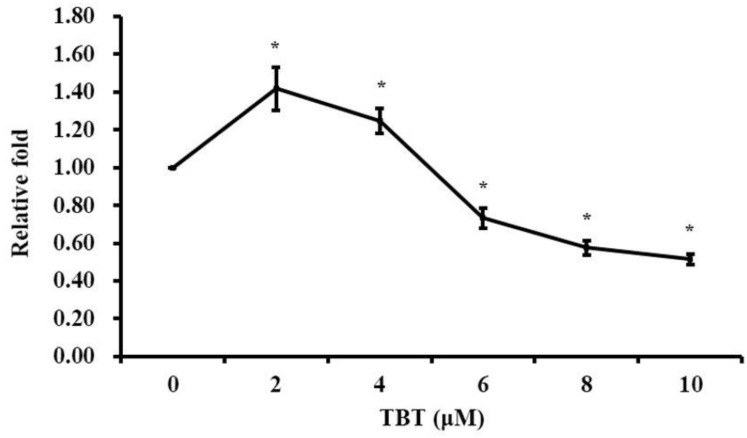
Cell proliferation after TBT exposure. The results are presented as the $\bar{X}\pm S$ of five independent experiments. The asterisks indicate the level of statistical significance (* *p* < 0.05) in comparison with the control group.

As shown in Figure 1, the cell proliferation dramatically increased after 2 or 4 μM of TBT treatment for 2 h. When the concentration of TBT was 6 μM or above, the cell proliferation decreased significantly. Therefore, in the following iTRAQ experiment, 0, 2, 6, and 10 μM TBT were selected as the control group, low-dose group, medium-dose group, and high-dose group, respectively.

### 3.2. TBT-Induced Alteration of Cellular Ultrastructure

**Figure 2:**
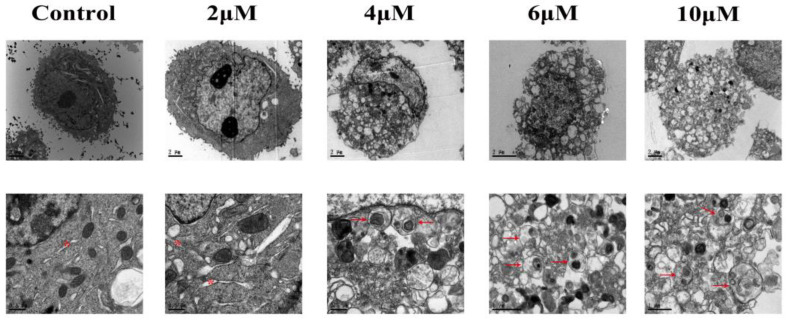
The effects of TBT on the cellular ultrastructure. The HL7702 cells were treated with different concentrations of TBT for 2 h, and the ultrastructure of the organelles changed significantly. Note the presence of ER stress (asterisks) and autophagosomes (arrow) in the TBT-treated cells.

In the control group, organelles such as the nucleus, mitochondria, ribosomes, endoplasmic reticulum (ER), and lysosomes had a normal morphology. In cells treated with 2 μΜ TBT, areas of the ER lumen were swollen, and ribosomal granules were being shed from the rough ER, indicating the activation of ER stress. In the 4 μΜ group, many autophagosomes appeared in the cytoplasm. As the concentration raised to 6 μΜ, the outer membrane of autophagosomes fused with the lysosomes, forming autolysosomes. In addition, typical morphologic hallmarks of apoptosis including cell shrinkage, nuclear condensation, membrane blebbing, and fragmentation were observed. When the concentration increased to 8 μΜ or above, the normal cell structure was completely disrupted (Figure 2).

### 3.3. TBT-Induced ER Stress

**Figure 3:**
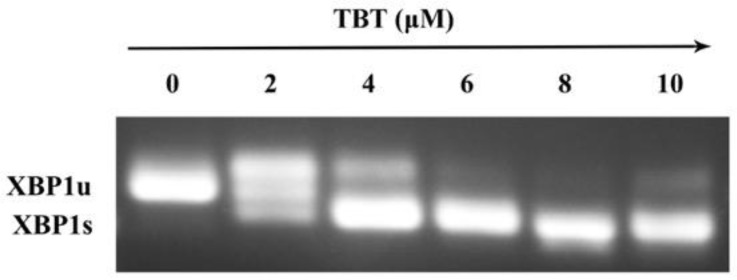
TBT-induced splicing of *XBP1* mRNA: *XBP1u* (unspliced *XBP1*) and *XBP1s* (spliced *XBP1*).

ER stress was measured by *XBP1* mRNA splicing. As shown in Figure 3, when the cells were exposed to 2 μΜ TBT, the *XBP1* mRNA was spliced into two bands, *XBP1u* (unsliced *XBP1*) and *XBP1s* (spliced *XBP1*), which indicated that ER stress occurred at 2 μΜ TBT. *XBP1* was completely spliced when the concentration reached 6 μΜ.

### 3.4. TBT-Induced Autophagy

**Figure 4:**
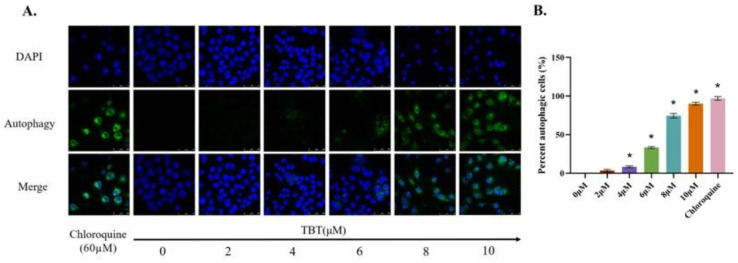
TBT-induced cells underwent autophagy. (**A**) Autophagic cells were observed under a confocal microscope. (**B**) The percentage of autophagic cells was estimated by counting cells in five randomly selected areas in duplicate (* *p* < 0.05).

Consistent with the results of the TEM, when the cells were treated with 4 μΜ or higher, the autophagic rates increased significantly in a concentration-dependent manner (Figure 4).

### 3.5. TBT-Induced Apoptosis

**Figure 5:**
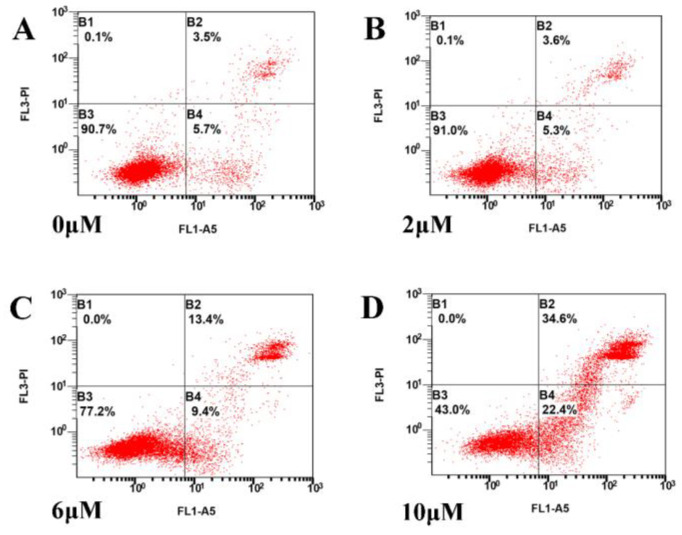
TBT-induced cells underwent apoptosis. (**A**) Control cells; cells exposed to (**B**) 2 μΜ, (**C**) 6 μΜ, and (**D**) 10 μΜ TBT. Exposure to TBT at concentrations of 6 μΜ and above significantly induced apoptosis, with a higher proportion of late apoptotic cells in the higher dose groups.

Flow cytometry revealed that a low dose of TBT (2 μΜ) did not induce apoptosis, while medium (6 μΜ) and high doses (10 μΜ) of TBT induced apoptosis significantly, which was consistent with the results of the MTT assay. Furthermore, there were more late apoptotic cells in the high-dose group than in the medium-dose group (Figure 5).

### 3.6. DEPs in Cells Exposed to TBT

**Figure 6:**
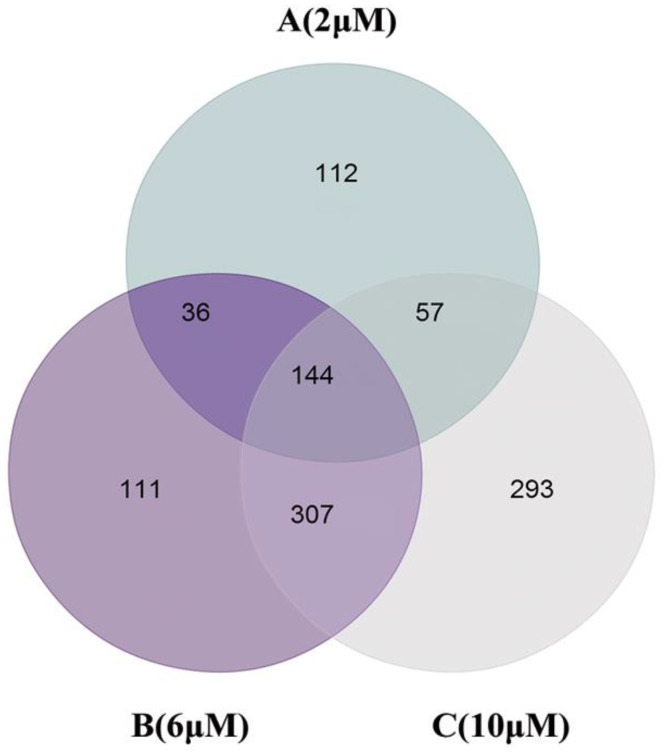
Venn diagram showing the overlap between the DEPs in the different groups.

The whole protein expression profiling was carried out to describe the hepatotoxicity of TBT by iTRAQ. Finally, 5112 high-confidence proteins (FDR ≤ 1%, unused protein score ≥1.3, and unique peptide number ≥2) were identified. According to the threshold criteria (*p* < 0.05 and fold change ≥1.5 or ≤0.65), 349, 598, and 801 DEPs were identified for the low-, medium-, and high-dose groups, respectively (Figure 6).

### 3.7. Protein Expression Profiles of HL7702 Cells with Low-Dose TBT Exposure

**Figure 7:**
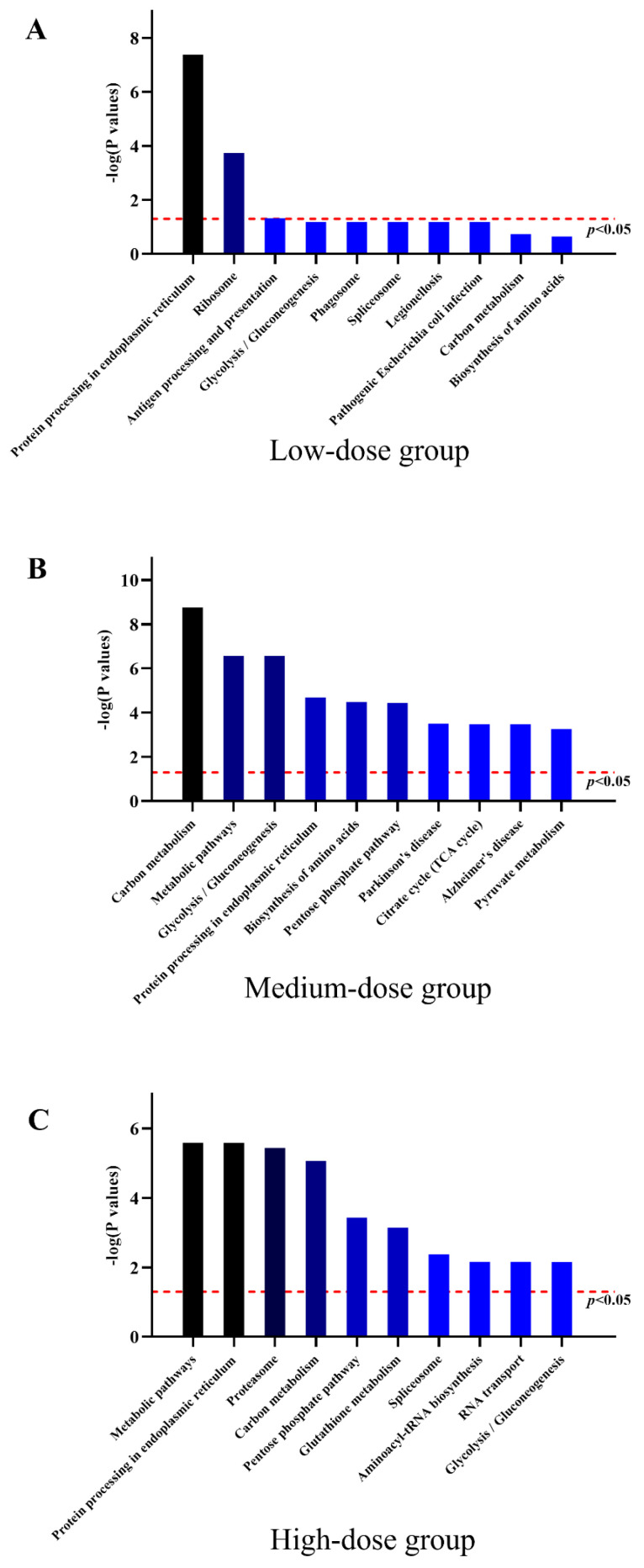
The top 10 KEGG pathways enriched with the DEPs in the (**A**) low-dose group, (**B**) medium-dose group, and (**C**) high-dose group. The bars indicate the -log (*p* values).

A low dose of TBT exposure affected the expression of 349 proteins in hepatocytes: 222 were upregulated, and 127 were downregulated. Three KEGG pathways—protein processing in ER, ribosome, and antigen processing and presentation (Figure 7A)—were found to be significantly enriched with an adjusted *p* value of <0.05 and contained at least three DEPs.

### 3.8. Protein Expression Profiles of HL7702 Cells with Medium-Dose TBT Exposure

A medium dose of TBT exposure affected the expression of 598 proteins: 312 were upregulated, and 286 were downregulated. Twenty-five KEGG pathways were found to be significantly enriched with an adjusted *p* value of <0.05 and contained at least three DEPs. The top 10 signaling pathways were carbon metabolism, metabolic pathways, glycolysis/gluconeogenesis, protein processing in ER, biosynthesis of amino acids, pentose phosphate pathway, Parkinson’s disease, citrate cycle (TCA cycle), Alzheimer’s disease, and pyruvate metabolism (Figure 7B).

### 3.9. Protein Expression Profiles of HL7702 Cells with High-Dose TBT Exposure

A high dose of TBT exposure affected the expression of 801 proteins: 385 were upregulated, and 416 were downregulated. Thirty-five KEGG pathways were found to be significantly enriched with an adjusted *p* value of <0.05 and contained at least three DEPs. The results revealed that the top 10 signaling pathways were metabolic pathways, protein processing in ER, proteasome, carbon metabolism, pentose phosphate pathway, glutathione metabolism, spliceosome, aminoacyl-tRNA biosynthesis, RNA transport, and glycolysis/gluconeogenesis (Figure 7C).

### 3.10. Effects of TBT on the Expression Levels of ER Stress-Associated Proteins

**Figure 8:**
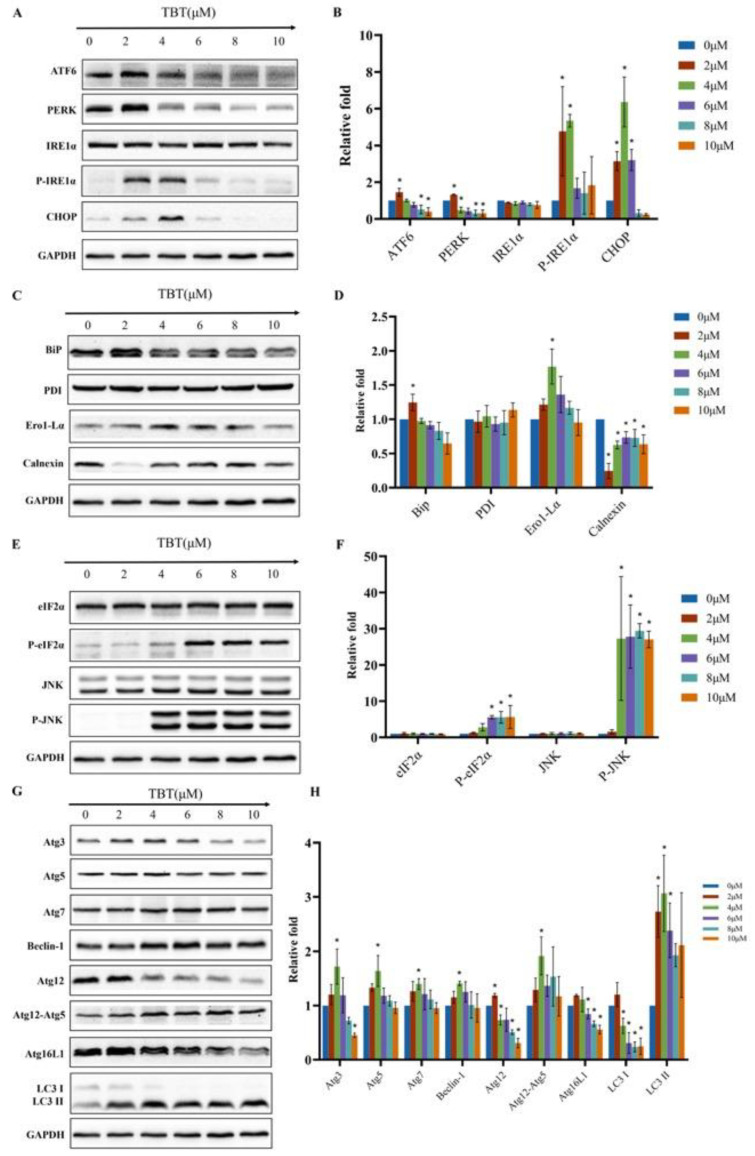
The effects of TBT on the expression levels of the primary ER stress-related and autophagy-related proteins. After exposure to TBT for 2 h, the expression levels of the ER stress-related and autophagy-related proteins were measured by Western blotting. (**A**,**C**,**E**,**G**) Representative autoradiographs of the ER stress-related proteins and autophagy-related proteins. (**B**,**D**,**F**,**H**) The intensity of each protein band was quantified by densitometry, and the values were normalized to the GAPDH value. The results are presented as the $\bar{X}\pm S$ of three independent experiments. The asterisks indicate the level of statistical significance (* *p* < 0.05) compared with the control value.

Since TBT can induce ER stress, the ER stress-associated proteins were further examined. Under exposure to 2 μΜ TBT, the levels of PERK, ATF6, BiP, and CHOP increased significantly, as shown in Figure 8A–F. Although the level of IRE1α remained unchanged, the P-IRE1α protein level and the P-IRE1α/IRE1α ratio increased significantly. The levels of PDI and Ero1-Lα increased slightly; however, the differences were not statistically significant. In contrast, the Calnexin level decreased under treatment with 2 μM TBT. When the TBT concentration increased to 4 μΜ, the level of PERK decreased, while the levels of P-eIF2α and Ero1-Lα increased.

### 3.11. Effects of TBT on the Expression Levels of Autophagy-Associated Proteins

Since TBT can cause autophagy, the autophagy-associated proteins were further examined. As shown in Figure 8G,H, under treatment with 4 μΜ TBT, the expression levels of Beclin1, Atg3, Atg5, Atg7, and Atg12-Atg5 (55 kDa) increased significantly, while the level of Atg12 (16 kDa) decreased. As the concentration of TBT increased, the levels of most of the autophagy-associated proteins began to decrease. We also evaluated the level of microtubule-associated protein light chain 3 (LC3) Ⅰ/Ⅱ, the marker of autophagy. The level of LC3Ⅱ increased under exposure to 2 μΜ TBT, while the level of LC3Ⅰ decreased under exposure to 4 μΜ TBT.

## 4. Discussion

Recently, high-throughput technologies have created new opportunities for toxicology research. The combination of multiomic and bioinformatics approaches enables researchers to identify the potential molecular mechanisms of toxic effects more quickly from different biological levels [39]. Previously, we used the transcriptomic method to survey the impacts of TBT on gene expression in hepatocytes and identified that the apoptotic pathway might be one of the dominant mechanisms of TBT-induced hepatotoxicity. Further study has indicated that the genes encoding tumor necrosis factor receptors, heat shock proteins, and kinases mediate TBT-induced apoptosis [20]. Considering that alterations in the mRNA levels may not ultimately affect protein abundance and function, proteomics, which aims to catalog and characterize the total complement of protein isoforms in cells, may provide more information [40]. Therefore, we used iTRAQ technology in this study to investigate the protein expression profile of hepatocytes exposed to TBT. These results indicated that “protein processing in ER” was among the top pathways, suggesting that in addition to apoptosis, ER stress might also be one of the major mechanisms of TBT-induced hepatotoxicity.

Interestingly, the TEM results showed that with increasing TBT concentration, ER swelling, autophagosomes, autolysosomes, and hallmarks of apoptosis appeared successively in the cells. Collectively considering the results of the MTT assay, transcriptomic analysis, proteomic analysis, and other specific experiments to detect ER stress, autophagy, or apoptosis, it is quite clear that low-dose (2 μΜ) TBT exposure causes unfolded protein response (UPR) and ER stress; as the exposure concentration increased, cells began to combat the damage through autophagy, and finally, severe damage initiated the process of apoptosis.

The ER is the membranous intracellular organelle that controls various aspects of protein quality including their synthesis, posttranslational modification, folding, assembly, and delivery [41,42,43], and guarantees that only properly folded proteins can reach their intended locations [44]. ER stress will occur when unfolded or misfolded proteins accumulate [45]. At this point, cells activate the UPR to buffer ER stress [46], which is mediated by three different but interrelated signal stress sensors: protein kinase R-like ER kinase (PERK), inositol-requiring protein 1α (IRE1α), and activating transcription factor 6 (ATF6) [47]. All of the above stress sensors are sequestered by binding immunoglobulin protein (BiP), also called glucose-regulated protein 78 (GRP78), in the unstressed state. However, when ER stress occurs, BiP binds to unfolded/misfolded proteins, which leads to the activation of IRE1α and PERK by autophosphorylation and ATF6 by proteolysis [48,49]. A previous study found that TBT could induce ER stress via Ca^2+^ depletion [50]; however, the roles of ATF6, PERK, and IRE1α are still unclear. Therefore, we further investigated eight ER stress-related proteins (ATF6, IRE1α, PERK, BiP, CHOP, Calnexin, Ero1-Lα, and PDI) selected by the KEGG pathway analysis and other important proteins including their phosphorylated forms (P-IRE1α, eIF2α, P-eIF2α, JNK and P-JNK) to elucidate the key role of ER stress in TBT-mediated hepatotoxicity.

PERK can inhibit protein translation and reduce protein synthesis to reduce the protein folding load of the ER [51]. Once activated, PERK phosphorylates the α-subunit of eukaryotic initiation factor 2 (eIF2α), reducing its mRNA translation while selectively enhancing the translation of certain mRNAs including CHOP and ATF4 [52,53]. In the present study, exposure to 2 μΜ TBT increased the PERK protein level, while exposure to 4 μΜ TBT decreased the PERK protein level. In addition, the level of phosphorylated eIF2α (P-eIF2α) was remarkably elevated after 6 μΜ of TBT exposure.

The stress-sensing domain of PERK has a clear correlation with that of IRE1 [54]. IRE1α has both kinase and Rnase activity. When ER stress occurs, IRE1α autophosphorylation stimulates its Rnase activity for noncanonical splicing of *XBP1* mRNA, producing the active transcription factor termed spliced *XBP1* [55]. Therefore, the splicing of *XBP1* is also recognized as an indicator of ER stress [55,56]. In the current study, the P-IRE1α protein level and the P-IRE1α/IRE1α ratio were significantly increased, and the *XBP1* mRNA splicing was initiated in the low-dose (2 μΜ) group, indicating the activation of ER stress.

ATF6 is initially anchored to the ER membrane under the normal state [57]. When various misfolded proteins accumulate in the ER, ATF6 is cleaved by proteases to produce the active fragment p50ATF6 after translocation to the Golgi apparatus, which then binds to stress response elements after translocation to the nucleus, where it activates ER-associated degradation (ERAD) and the transcription of genes encoding ER chaperones including GRP78 and protein disulfide isomerase (PDI) to enhance the folding capacity of ER [58,59]. In addition, ATF6 can activate the transcription of *XBP1* and ERAD genes [60]. Similar to PERK, in the present study, exposure to 2 μΜ TBT increased the ATF6 protein level, while exposure to 4 μΜ TBT decreased the ATF6 protein level.

The induction of ER chaperones to reduce the amount of unfolded proteins is another major UPR pathway [51]. GRP78/BiP is one of the best-characterized ER chaperones. Due to its ability to control the activation of ER stress, GRP78/BiP is regarded as a marker for ER stress [61]. PDI is another ER chaperone that can promote the formation of disulfide bonds and inhibit apoptosis. In the ER of human cells, Ero1-Lα is the main enzyme that catalyzes the formation of disulfide bonds [62]. Calnexin is also an important molecular chaperone in the ER for quality control, which recognizes the immature glycosylation state of membrane proteins and thus assists in folding newly synthesized proteins or degrading incorrectly folded proteins [63]. In the current study, the BiP level was elevated in the 2 μM group, but remained unaffected in the other dose groups. The levels of Ero1-Lα increased slightly after treatment with 4 μM TBT, however, the levels of PDI remained unchanged. In contrast to the GRP78 level, the Calnexin level decreased under treatment with 2 μM TBT while the other concentrations remained constant. Considering these results, we speculate that GRP78/BiP is the dominant chaperone involved in TBT-induced ER stress.

All of the above results indicate that ER stress did occur in the hepatocytes after exposure to low dose (2 μΜ) TBT and that all three UPR pathways were involved in synergistically increasing the ER protein folding capacity as well as the degradation of unfolded/misfolded proteins. Nevertheless, when the stimulation is sustained or excessive, it is impossible for the UPR to restore the ER to its normal state, and the damaged ER may be engulfed and degraded by autophagic vesicles [64]. It has been reported that the PERK–eIF2α pathway and IRE1–JNK pathway play important roles in the association between ER stress and autophagy, but ATF6 may not be required for ER stress-induced autophagy [65]. Interestingly, in this study, the levels of P-IF2α and P-JNK were markedly elevated under treatment with 4 μΜ TBT, strongly suggesting that these two pathways mediate the induction of autophagy under severe ER stress.

Autophagy, as an evolutionarily conserved biological process, maintains cellular homeostasis and is strongly linked to ER stress and apoptosis [58]. The process of macroautophagy mainly starts with the response of stress signaling pathways by Beclin-1/VPS34 at the ER and other membranes. Then, Atg5 binds to Atg12 and noncovalently interacts with Atg16L to form a multimeric complex. When LC3-I is converted to LC3-II and inserted into the phagophore membrane, phagocytes engulf the degradation targets to form autophagosomes. Finally, autophagosomes fuse with lysosomes for degradation [66]. Therefore, Atg5–Atg12–Atg16L1 and LC3/Atg8-phosphatidyl ethanolamine (PE) are two ubiquitin-like protein complexes regulating the elongation and closure of autophagosomes [67]. In the Atg5–Atg12–Atg16L1 system, Atg7 activates Atg12 in an ATP-dependent manner, similar to the E1 ubiquitin-activating enzyme. Atg12 is then transferred to Atg10, which is an E2-like ubiquitin carrier protein that enhances the covalent attachment to form the irreversible Atg12–Atg5 conjugate (55 kDa). The conjugated Atg5–Atg12 complex pairs with Atg16L dimers to form the multimeric Atg5–Atg12–Atg16L complex, which is required for autophagosome formation [68,69,70]. In the LC3/Atg8-PE system, LC3 is proteolytically cleaved by Atg4 to produce LC3-I. LC3-I and PE are then conjugated via ubiquitination-like reactions activated by Atg7 and Atg3 to generate processed LC3-II [71,72]. Interestingly, consistent with the TEM results (autophagosomes were observed when cells were exposed to 4 μΜ TBT), the protein levels of Atg3, Atg7, Atg12, Atg12-Atg5, Atg16L1, and LC3-II were significantly increased under this exposure concentration. Therefore, these results indicate that the canonical macroautophagy pathway is involved in TBT-induced autophagy.

Numerous studies have revealed that TBT can induce apoptosis in vivo and in vitro [73,74]. Our previous study also confirmed that after treatment with 6 μΜ TBT, the HL7702 cells underwent significant apoptosis [20]. Subsequent transcriptomic analysis further revealed that TBT-induced apoptosis was mediated by the Bcl-2 family, HSP family, mitogen-activated protein kinase (MAPK) family, and tumor necrosis factor (TNF) receptor superfamily through the death receptor, mitochondrial, and ER pathways [20]. In the current study, we speculated that autophagy is the balancing point that determines whether the cell fate is survival or death after TBT exposure; if autophagy cannot eliminate the damage, the cell is destroyed by apoptosis.

## 5. Conclusions

**Figure 9:**
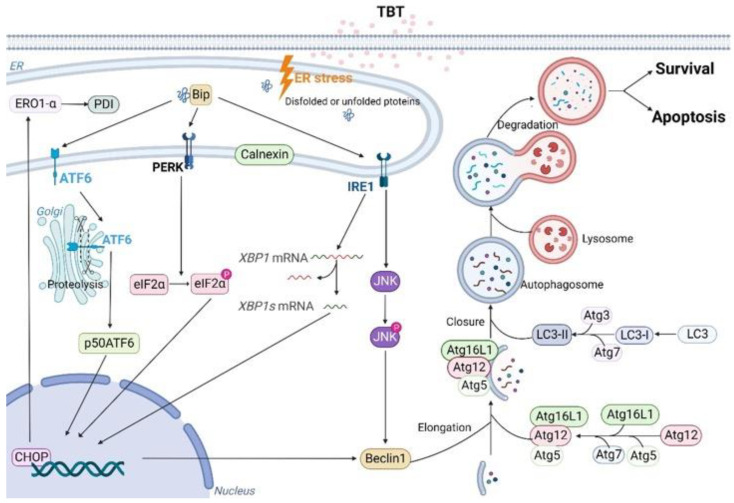
Schematic diagram of TBT-induced hepatotoxicity. Low dose (2 μΜ) TBT exposure induced ER stress and UPR activation. When the exposure concentration was increased to 4 μΜ, the UPR failed to restore the ER to its normal state and autophagy was induced. As the concentration of TBT increased, autophagy was unable to repair the damage, and the cells eventually underwent apoptosis.

In conclusion, this study indicates that exposure to low dose (2 μΜ) TBT induced ER stress and UPR activation through all three ER receptors (PERK, ATF6, and IRE1) and the related chaperones to enhance the folding capacity of the ER and degrade unfolded/misfolded proteins. When the exposure concentration increased to 4 μΜ, the UPR could not restore the ER to its normal state, autophagy was triggered, and the damaged organelles were engulfed by autophagosomes. When the TBT concentration continued to increase, autophagy could not eliminate the damage, and the cells finally underwent apoptosis (Figure 9). Due to the use of a single liver cell line, caution should still be taken and more in vitro and in vivo experiments are required to identify more specific pathways.

## Data Availability

The data that support the findings of this study are available from the corresponding author upon reasonable request.

## References

[1] Furdek M., Vahčič M., Ščančar J., Milačič R., Kniewald G., Mikac N. (2012). Organotin compounds in seawater and *Mytilus galloprovincialis* mussels along the Croatian Adriatic Coast. Mar. Pollut. Bull..

[2] Okoro H.K., Fatoki O.S., Adekola F.A., Ximba B.J., Snyman R.G., Opeolu B. (2011). Human exposure, biomarkers, and fate of organotins in the environment. Rev. Environ. Contam. Toxicol..

[3] Antizar-Ladislao B. (2008). Environmental levels, toxicity and human exposure to tributyltin (TBT)-contaminated marine environment. A review. Environ. Int..

[4] Furdek M., Mikac N., Bueno M., Tessier E., Cavalheiro J., Monperrus M. (2016). Organotin persistence in contaminated marine sediments and porewaters: In situ degradation study using species-specific stable isotopic tracers. J. Hazard. Mater..

[5] Gipperth L. (2009). The legal design of the international and European Union ban on tributyltin antifouling paint: Direct and indirect effects. J. Environ. Manag..

[6] Omae I. (2003). Organotin Antifouling Paints and Their Alternatives. ChemInform.

[7] Horiguchi T. (2006). Masculinization of female gastropod mollusks induced by organotin compounds, focusing on mechanism of actions of tributyltin and triphenyltin for development of imposex. Environ. Sci. Int. J. Environ. Physiol. Toxicol..

[8] Marshall D.J., Rajkumar A. (2003). Imposex in the indigenous *Nassarius kraussianus* (Mollusca: Neogastropoda) from South African harbours. Mar. Pollut. Bull..

[9] Hagger J.A., Depledge M.H., Galloway T.S. (2005). Toxicity of tributyltin in the marine mollusc *Mytilus edulis*. Mar. Pollut. Bull..

[10] Hoch M. (2001). Organotin compounds in the environment—An overview. Appl. Geochem..

[11] Viglino L., Pelletier E., St-Louis R. (2004). Highly persistent butyltins in northern marine sediments: A long-term threat for the Saguenay Fjord (Canada). Environ. Toxicol. Chem..

[12] Yi A.X., Leung K.M.Y., Lam M.H.W., Lee J.-S., Giesy J.P. (2012). Review of measured concentrations of triphenyltin compounds in marine ecosystems and meta-analysis of their risks to humans and the environment. Chemosphere.

[13] Santos M.M., Enes P., Reis-Henriques M.A., Kuballa J., Castro L.F.C., Vieira M.N. (2009). Organotin levels in seafood from Portuguese markets and the risk for consumers. Chemosphere.

[14] Rantakokko P., Turunen A., Verkasalo P.K., Kiviranta H., Männistö S., Vartiainen T. (2008). Blood levels of organotin compounds and their relation to fish consumption in Finland. Sci. Total Environ..

[15] Strand J., Jacobsen J.A. (2005). Accumulation and trophic transfer of organotins in a marine food web from the Danish coastal waters. Sci. Total Environ..

[16] De Lemos Barbosa C.M., Ferrão F.M., Graceli J.B. (2018). Organotin Compounds Toxicity: Focus on Kidney. Front. Endocrinol..

[17] Grondin M., Marion M., Denizeau F., Averill-Bates D.A. (2007). Tributyltin induces apoptotic signaling in hepatocytes through pathways involving the endoplasmic reticulum and mitochondria. Toxicol. Appl. Pharmacol..

[18] Suzuki J.S., Ishido M. (2011). Transcriptome of tributyltin-induced apoptosis of the cultured rat mesencephalic neural stem cells. Toxicology.

[19] Tu W.-W., Ji L.-D., Qian H.-X., Zhou M., Zhao J.-S., Xu J. (2016). Tributyltin induces disruption of microfilament in HL7702 cells via MAPK-mediated hyperphosphorylation of VASP. Environ. Toxicol..

[20] Zhou M., Feng M., Fu L.-L., Ji L.-D., Zhao J.-S., Xu J. (2016). Toxicogenomic analysis identifies the apoptotic pathway as the main cause of hepatotoxicity induced by tributyltin. Food Chem. Toxicol. Int. J. Publ. Br. Ind. Biol. Res. Assoc..

[21] Burton E.D., Phillips I.R., Hawker D.W. (2004). Sorption and desorption behavior of tributyltin with natural sediments. Environ. Sci. Technol..

[22] Ciesielski T., Wasik A., Kuklik I., Skóra K., Namieśnik J., Szefer P. (2004). Organotin compounds in the liver tissue of marine mammals from the Polish coast of the Baltic Sea. Environ. Sci. Technol..

[23] Dos Santos D.M., Santos G.S., Cestari M.M., de Oliveira Ribeiro C.A., de Assis H.C.S., Yamamoto F., Guiloski I.C., de Marchi M.R.R., Montone R.C. (2014). Bioaccumulation of butyltins and liver damage in the demersal fish Cathorops spixii (*Siluriformes*, *Ariidae*). Environ. Sci. Pollut. Res. Int..

[24] Nielsen J.B., Strand J. (2002). Butyltin compounds in human liver. Environ. Res..

[25] Senthilkumar K., Duda C.A., Villeneuve D.L., Kannan K., Falandysz J., Giesy J.P. (1999). Butyltin compounds in sediment and fish from the Polish Coast of the Baltic Sea. Environ. Sci. Pollut. Res. Int..

[26] Takahashi S., Mukai H., Tanabe S., Sakayama K., Miyazaki T., Masuno H. (1999). Butyltin residues in livers of humans and wild terrestrial mammals and in plastic products. Environ. Pollut..

[27] Chapman R.W., Guillette L.J. (2013). Contaminants and impoSEX: Transcriptomics of contaminant-induced sex change. Mol. Ecol..

[28] Lam N.H., Jeong H.-H., Kang S.-D., Kim D.-J., Ju M.-J., Horiguchi T., Cho H.-S. (2017). Organotins and new antifouling biocides in water and sediments from three Korean Special Management Sea Areas following ten years of tributyltin regulation: Contamination profiles and risk assessment. Mar. Pollut. Bull..

[29] Tryphonas H., Cooke G., Caldwell D., Bondy G., Parenteau M., Hayward S., Pulido O. (2004). Oral (gavage), in utero and post-natal exposure of Sprague-Dawley rats to low doses of tributyltin chloride. Part II: Effects on the immune system. Food Chem. Toxicol. Int. J. Publ. Br. Ind. Biol. Res. Assoc..

[30] Huang C.F., Yang C.Y., Tsai J.R., Wu C.T., Liu S.H., Lan K.C. (2018). Low-dose tributyltin exposure induces an oxidative stress-triggered JNK-related pancreatic β-cell apoptosis and a reversible hypoinsulinemic hyperglycemia in mice. Sci. Rep..

[31] Katika M.R., Hendriksen P.J.M., van Loveren H., Peijnenburg A. (2011). Exposure of Jurkat cells to bis (tri-n-butyltin) oxide (TBTO) induces transcriptomics changes indicative for ER- and oxidative stress, T cell activation and apoptosis. Toxicol. Appl. Pharmacol..

[32] Krug H.F. (2012). Caspase-10 is the key initiator caspase involved in tributyltin-mediated apoptosis in human immune cells. J. Toxicol..

[33] Wang Y., Wang S., Luo X., Yang Y., Jian F., Wang X., Xie L. (2014). The roles of DNA damage-dependent signals and MAPK cascades in tributyltin-induced germline apoptosis in Caenorhabditis elegans. Chemosphere.

[34] Kumar P., Nagarajan A., Uchil P.D. (2018). Analysis of Cell Viability by the MTT Assay. Cold Spring Harb. Protoc..

[35] Smith P.K., Krohn R.I., Hermanson G.T., Mallia A.K., Gartner F.H., Provenzano M.D., Fujimoto E.K., Goeke N.M., Olson B.J., Klenk D.C. (1985). Measurement of protein using bicinchoninic acid. Anal. Biochem..

[36] Wiśniewski J.R., Zougman A., Nagaraj N., Mann M. (2009). Universal sample preparation method for proteome analysis. Nat. Methods.

[37] Liu X., Wang J., Gao L., Liu H., Liu C. (2017). iTRAQ-Based Proteomic Analysis of Neonatal Kidney from Offspring of Protein Restricted Rats Reveals Abnormalities in Intraflagellar Transport Proteins. Cell. Physiol. Biochem. Int. J. Exp. Cell. Physiol. Biochem. Pharmacol..

[38] Cox D.J., Strudwick N., Ali A.A., Paton A.W., Paton J.C., Schröder M. (2011). Measuring signaling by the unfolded protein response. Methods Enzym..

[39] Brinke A., Buchinger S. (2017). Toxicogenomics in Environmental Science. Adv. Biochem. Eng. Biotechnol..

[40] Perkel J.M. (2021). Single-cell proteomics takes centre stage. Nature.

[41] Hetz C., Papa F.R. (2018). The Unfolded Protein Response and Cell Fate Control. Mol. Cell.

[42] Ron D., Walter P. (2007). Signal integration in the endoplasmic reticulum unfolded protein response. Nat. Rev. Mol. Cell Biol..

[43] Shen X., Zhang K., Kaufman R.J. (2004). The unfolded protein response—A stress signaling pathway of the endoplasmic reticulum. J. Chem. Neuroanat..

[44] Stefan C.J., Manford A.G., Baird D., Yamada-Hanff J., Mao Y., Emr S.D. (2011). Osh proteins regulate phosphoinositide metabolism at ER-plasma membrane contact sites. Cell.

[45] Ochoa C.D., Wu R.F., Terada L.S. (2018). ROS signaling and ER stress in cardiovascular disease. Mol. Asp. Med..

[46] Almanza A., Carlesso A., Chintha C., Creedican S., Doultsinos D., Leuzzi B., Luís A., McCarthy N., Montibeller L., More S. (2019). Endoplasmic reticulum stress signalling—From basic mechanisms to clinical applications. FEBS J..

[47] Marciniak S.J., Chambers J.E., Ron D. (2022). Pharmacological targeting of endoplasmic reticulum stress in disease. Nat. Rev. Drug Discov..

[48] Bertolotti A., Zhang Y., Hendershot L.M., Harding H.P., Ron D. (2000). Dynamic interaction of BiP and ER stress transducers in the unfolded-protein response. Nat. Cell Biol..

[49] Oikawa D., Kimata Y., Kohno K., Iwawaki T. (2009). Activation of mammalian IRE1alpha upon ER stress depends on dissociation of BiP rather than on direct interaction with unfolded proteins. Exp. Cell Res..

[50] Isomura M., Kotake Y., Masuda K., Miyara M., Okuda K., Samizo S., Sanoh S., Hosoi T., Ozawa K., Ohta S. (2013). Tributyltin-induced endoplasmic reticulum stress and its Ca(2+)-mediated mechanism. Toxicol. Appl. Pharmacol..

[51] Lai E., Teodoro T., Volchuk A. (2007). Endoplasmic reticulum stress: Signaling the unfolded protein response. Physiology.

[52] Brewer J.W., Diehl J.A. (2000). PERK mediates cell-cycle exit during the mammalian unfolded protein response. Proc. Natl. Acad. Sci. USA.

[53] Harding H.P., Zhang Y., Ron D. (1999). Protein translation and folding are coupled by an endoplasmic-reticulum-resident kinase. Nature.

[54] Liu C.Y., Schröder M., Kaufman R.J. (2000). Ligand-independent dimerization activates the stress response kinases IRE1 and PERK in the lumen of the endoplasmic reticulum. J. Biol. Chem..

[55] Yoshida H., Matsui T., Yamamoto A., Okada T., Mori K. (2001). XBP1 mRNA is induced by ATF6 and spliced by IRE1 in response to ER stress to produce a highly active transcription factor. Cell.

[56] Oishi N., Duscha S., Boukari H., Meyer M., Xie J., Wei G., Schrepfer T., Roschitzki B., Boettger E.C., Schacht J. (2015). XBP1 mitigates aminoglycoside-induced endoplasmic reticulum stress and neuronal cell death. Cell Death Dis..

[57] Malhi H., Kaufman R.J. (2011). Endoplasmic reticulum stress in liver disease. J. Hepatol..

[58] Song S., Tan J., Miao Y., Li M., Zhang Q. (2017). Crosstalk of autophagy and apoptosis: Involvement of the dual role of autophagy under ER stress. J. Cell. Physiol..

[59] Wang M., Kaufman R.J. (2016). Protein misfolding in the endoplasmic reticulum as a conduit to human disease. Nature.

[60] Walter P., Ron D. (2011). The unfolded protein response: From stress pathway to homeostatic regulation. Science.

[61] Lee A.S. (2005). The ER chaperone and signaling regulator GRP78/BiP as a monitor of endoplasmic reticulum stress. Methods.

[62] Chu Y., Yang C., Chen X., Zheng W., Yang Y., Tang Y. (2009). Structure-function analysis of human protein Ero1-Lalpha. Biochem. Biophys. Res. Commun..

[63] Brady A.E., Limbird L.E. (2002). G protein-coupled receptor interacting proteins: Emerging roles in localization and signal transduction. Cell. Signal..

[64] Qi Z., Chen L. (2019). Endoplasmic Reticulum Stress and Autophagy. Adv. Exp. Med. Biol..

[65] Ding W.X., Ni H.M., Gao W., Yoshimori T., Stolz D.B., Ron D., Yin X.M. (2007). Linking of autophagy to ubiquitin-proteasome system is important for the regulation of endoplasmic reticulum stress and cell viability. Am. J. Pathol..

[66] Glick D., Barth S., Macleod K.F. (2010). Autophagy: Cellular and molecular mechanisms. J. Pathol..

[67] He C., Klionsky D.J. (2009). Regulation mechanisms and signaling pathways of autophagy. Annu. Rev. Genet..

[68] Hanada T., Noda N.N., Satomi Y., Ichimura Y., Fujioka Y., Takao T., Inagaki F., Ohsumi Y. (2007). The Atg12-Atg5 conjugate has a novel E3-like activity for protein lipidation in autophagy. J. Biol. Chem..

[69] Li S.-P., He J.-D., Wang Z., Yu Y., Fu S.-Y., Zhang H.-M., Zhang J.-J., Shen Z.-Y. (2016). miR-30b inhibits autophagy to alleviate hepatic ischemia-reperfusion injury via decreasing the Atg12-Atg5 conjugate. World J. Gastroenterol..

[70] Otomo C., Metlagel Z., Takaesu G., Otomo T. (2013). Structure of the human ATG12~ATG5 conjugate required for LC3 lipidation in autophagy. Nat. Struct. Mol. Biol..

[71] Yang Z.J., Chee C.E., Huang S., Sinicrope F.A. (2011). The role of autophagy in cancer: Therapeutic implications. Mol. Cancer Ther..

[72] Tooze S.A., Abada A., Elazar Z. (2014). Endocytosis and autophagy: Exploitation or cooperation?. Cold Spring Harb. Perspect. Biol..

[73] Ling L., Wen J., Tao L., Zhao M., Ge W., Wang L., Zhang J., Weng D. (2019). RIP1 and RIP3 contribute to Tributyltin-induced toxicity in vitro and in vivo. Chemosphere.

[74] Kulbay M., Johnson B., Bernier J. (2019). DNA fragmentation factor 40 expression in T cells confers sensibility to tributyltin-induced apoptosis. Toxicology.

